# Influence of rovibrational excitation on the non-diabatic state-to-state dynamics for the Li(2p) + H_2_ → LiH + H reaction

**DOI:** 10.1038/s41598-017-03274-y

**Published:** 2017-06-08

**Authors:** Di He, Jiuchuang Yuan, Maodu Chen

**Affiliations:** Key Laboratory of Materials Modification by Laser, Electron, and Ion Beams (Ministry of Education), School of Physics and Optoelectronic Technology, Dalian University of Technology, Dalian, 116024 P. R. China

## Abstract

The non-adiabatic state-to-state dynamics of the Li(2p) + H_2_ → LiH + H reaction has been studied using the time-dependent wave packet method, based on a set of diabatic potential energy surfaces recently developed by our group. Integral cross sections (ICSs) can be increase more than an order of magnitude by the vibrational excitation of H_2_, whereas the ICSs are barely affected by the rotational excitation of H_2_. Moreover, ICSs of the title reaction with vibrationally excited H_2_ decrease rapidly with increasing collision energy, which is a typical feature of non-threshold reaction. This phenomenon implies that the title reaction can transformed from an endothermic to an exothermic reaction by vibrational excitation of H_2_. With the increase of the collision energy, the sideways and backward scattered tendencies of LiH for the Li(2p) + H_2_(*v* = 0, *j* = 0, 1) → LiH + H reactions are enhanced slightly, while the backward scattering tendency of LiH for the Li(2p) + H_2_(*v* = 1, *j* = 0) → LiH + H reaction becomes remarkably weakened. For the reaction with vibrationally excited H_2_ molecule, both direct and indirect reaction mechanism exist simultaneously.

## Introduction

The reaction involving alkali atoms and H_2_ molecule have been studied for decades^1–22^. Most of the researches focused on reactive collisions between ground state alkali atoms and hydrogen molecules^1–10^. However, only a few studies were devoted to reactions between electronically excited alkali metal atoms and H_2_ molecules. The collision of excited alkali metal atoms with H_2_ molecules can lead to a non-reactive quenching process or a reactive process. A diabatic transition is involved in both kinds of processes. Various experimental techniques have been used for investigating both classes of reactions^12–14^. Bililign *et al*.^11^ applied the “half-collision” pump-probe technique to measure the far-wing absorption profiles of NaH_2_ collision complex in the Na(4^2^P) + H_2_ reaction. In this experiment, the rotational state distributions of product NaH were observed. The Cs(6D_3/2_) + H_2_ → CsH + H reaction was studied in a crossed-beam experiment and CsH product was detected by laser-induced fluorescence^12^. Reaction cross sections and the rotational state distributions of CsH molecules were determined at the collision energy of 0.09 eV. Furthermore, the K(5p^2^P) + H_2_ → KH + H reaction was investigated by scattering state spectroscopic^13^. Rovibrational states populations of the KH product were determined from far-wing absorption spectra. The product nascent quantum state distributions of the Rb(5^2^D, 7^2^S) + H_2_ → RbH + H reaction were determined by a laser pump-probe technique^14^.

The Li + H_2_ reaction is the simplest chemical reaction within this class of reactions which only involves an alkali atom and hydrogen molecule. Therefore, it is an ideal candidate for studying the reaction dynamics of this class of reactions. In 1987, Myers *et al*.^15^ measured cross section of the Li(2p) + H_2_ → LiH + H reaction at 788 K using absorption techniques. The quenching process during the Li(3p) + H_2_ → Li(3s) + H_2_ was investigated under gas cell conditions^16^. Bililign *et al*.^17^ studied the Li(2p) + H_2_ → LiH + H reaction using the laser pump-probe far-wing scattering technique; the LiH product was observed when the collision energy was sufficiently high for the product formation. Furthermore, they found that reactive collisions take place preferentially in bent structure (near *C*_2*v*_ geometry). The influence of the vibrational excitation of H_2_ reactant on the Li(2p) + H_2_ → LiH + H reaction was studied by a pump-probe technique. Moreover, the rotational population distributions of LiH product were also obtained from the same experiment^18^. Up to now, the theoretical studies on the title reaction are still at a primary stage compared with the experimental studies. In 1997, Martinez performed a quantum mechanical nuclear dynamics for the Li(2p) + H_2_ reaction and found that the non-adiabatic process was ultrafast^19^. Based on the adiabatic potential energy surfaces (PESs) of Li(2p, 3s, 3p) + H_2_ system, Lee *et al*.^20^ predicted the essential features of these reactions. The geometries of intermediates during the Li(2p, 3s) + H_2_ reactions were examined in detail. In addition, they found that the reactive collision of the Li(2p) + H_2_ → LiH + H reaction mainly occurs through a bent (*C*_2*v*_) configuration. This conclusion is consistent with experimental results. However, the reaction dynamic calculations of the Li(2p) + H_2_ → LiH + H reaction have not been conducted in Lee’s study. Hsiao *et al*.^21^ carried quasi-classical trajectory calculations on the Li(2p) + H_2_ → LiH + H reaction based on two adiabatic PESs. The reactants correlate with the first exited state (2^2^A′) adiabatic PES and the products correlate with the ground state (1^2^A′) adiabatic PES. The influences of the vibrational excitation and collision energy on the title reaction were studied in their work. Moreover, the trajectories start from the first excited state 2^2^A′ and then hop to the ground state 1^2^A′ to form products at the exit channel. It should be noted that the transition probability between two adiabatic PESs was set to one, which is a simple approximation to treat the non-diabatic process during the Li(2p) + H_2_ → LiH + H reaction. Recently, our group presented a set of diabatic PESs (HYLC PESs) using a diabatization scheme based on the transition dipole momentum operators for the title reaction^22^. The influence of collision energy on the reaction probability, integral cross section (ICS) and differential cross section (DCS) were studied based on the HYLC PESs using the time dependent wave packet (TDWP) method^22^. In the current work, in order to get detailed information on the influence of rovibrational excitations on the Li(2p) + H_2_ → LiH + H reaction, the state-to-state dynamic of this reaction has been studied based on the HYLC PESs.

## Results

### Reaction probabilities

**Figure 1:**
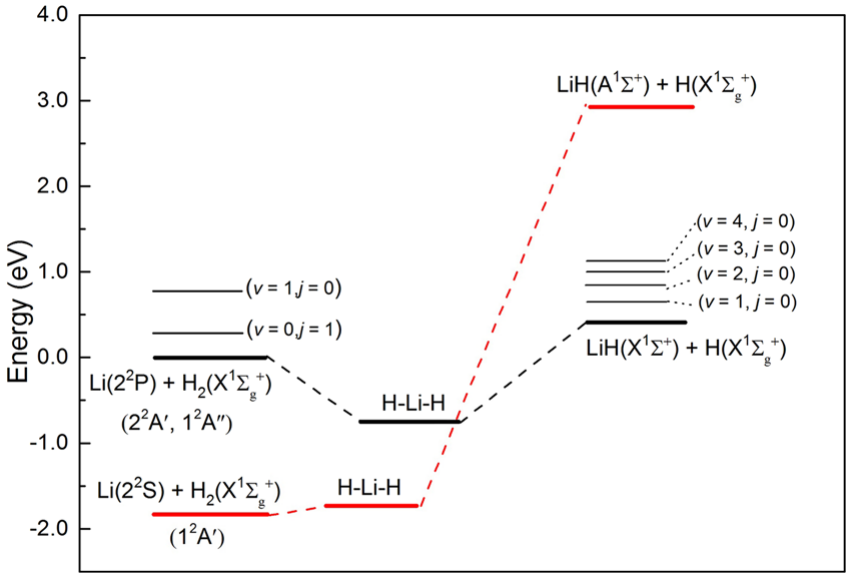
Schematic reaction path and energetic for the Li(2p) + H_2_ → LiH + H reaction on the HYLC diabatic PESs.

**Figure 2:**
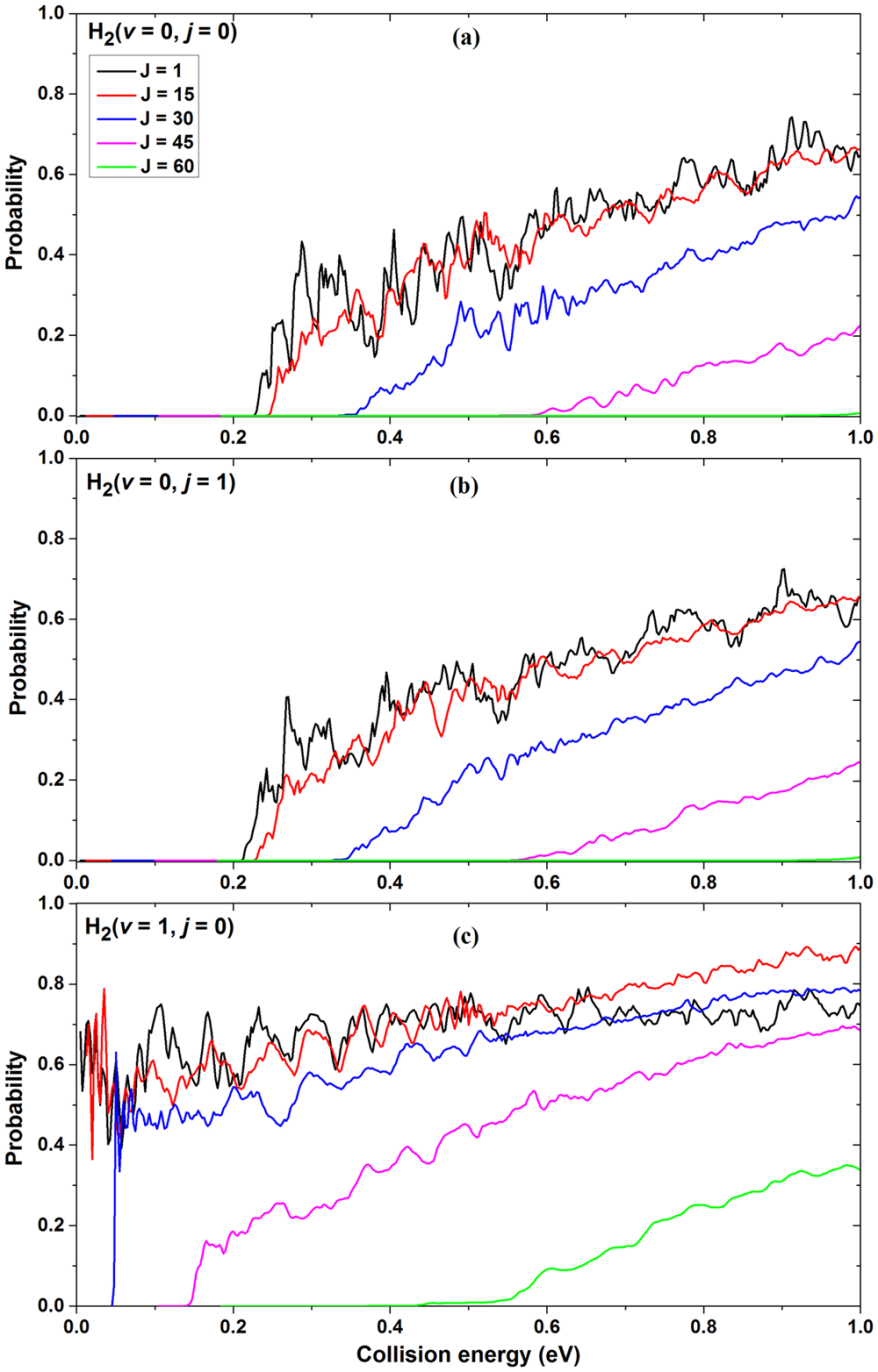
Reaction probabilities of the Li(2p) + H_2_ → LiH + H reaction with (a) H_2_(*v* = 0, *j* = 0), (b) H_2_(*v* = 0, *j* = 1) and (c) H_2_(*v* = 1, *j* = 0) as a function of collision energy.

In order to conveniently analyze dynamic results within the framework of PESs, the schematic energy diagram of the title reaction is presented in Fig. 1 according to the HYLC PESs. As can be seen from Fig. 1, the energy of Li(2p) + H_2_(*v* = 1, *j* = 0) is higher than the that of Li(2p) + H_2_(*v* = 0, *j* = 1). The reaction probabilities of the reaction with different rovibrational states of the H_2_ molecule and total angular momentum quantum numbers are depicted in Fig. 2 as a function of the collision energy. The three panels of Fig. 2 from (a) to (c) correspond to different rovibrational states of the reactant molecule at H_2_(*v* = 0, *j* = 0), H_2_(*v* = 0, *j* = 1), and H_2_(*v* = 1, *j* = 0) (hereinafter the same for the similar pictures). It can be seen that the reaction probabilities in Fig. 2(a) are similar to that in Fig. 2(b). In addition, it can be concluded that the reaction threshold of the Li + H_2_(*v* = 0, *j* = 0) reaction is slightly higher than that of Li + H_2_(*v* = 0, *j* = 1) reaction. As can be seen from the bottom panel of Fig. 2, the thresholds of the Li(2p) + H_2_ → LiH + H reaction for total angular momentum quantum number J = 1 and J = 15 no longer exists when the reactant molecule is excited to the first vibrational excitation state.

The behavior of the reaction probability indicates that the rotational excitation has a small effect on the reactivity, while the vibrational excitation has a great influence on it. This is due to the fact that the energy released from the excited vibrational state H_2_(*v* = 1, *j* = 0) is much higher than the one which is released by the excited rotational state H_2_(*v* = 0, *j* = 1). It can be seen that the oscillations for the reactions with small total angular momentum are stronger than the one for the reactions with large total angular momentum. The reactions with small angular momentum prefer to take place in *C*_2v_ geometry (H-Li-H), which means the reactions are dominated by insertion mechanism. However, for the reactions with large total angular momentum, these reactions are dominated by abstraction mechanism. Moreover, we note that as the collision energies increase, the oscillations for reactions with small total angular momentum become unapparent. In all cases, the centrifugal barrier makes the threshold larger with the increase of the total angular momentum quantum number J.

### Reaction cross sections

**Figure 3:**
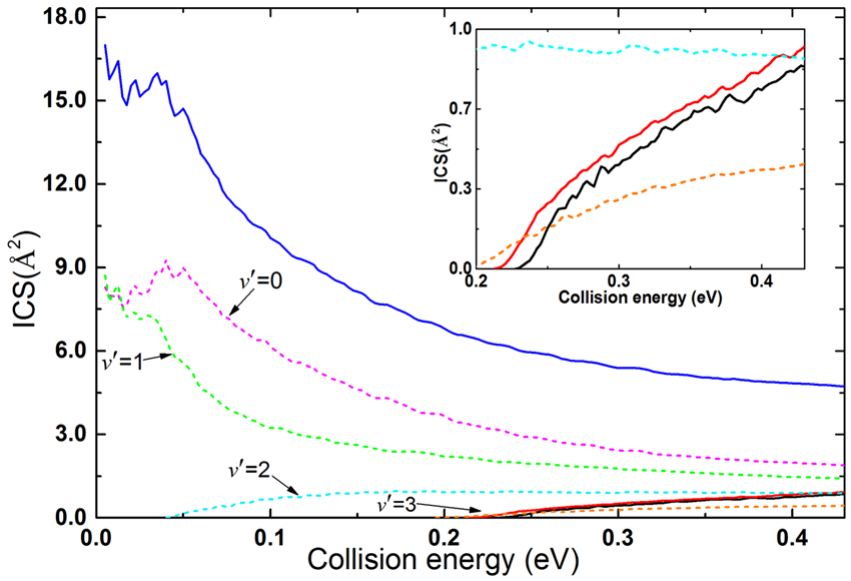
Total ICSs of the Li(2p) + H_2_ → LiH + H reaction for different initial rovibrational states of H_2_ molecule and product vibrationally resolved ICSs of the Li(2p) + H_2_(*v* = 1, *j* = 0) → LiH(*v*′ = 0, 1, 2, 3) + H reaction. Black, red and blue solid lines stand for H_2_(*v* = 0, *j* = 0), H_2_ (*v* = 0, *j* = 1) and H_2_(*v* = 1, *j* = 0), respectively. Magenta, green, cyan and orange dash lines stand for *v*′ = 0, 1, 2 and 3 respectively.

In the TDWP calculations, the maximum of total angular momentum quantum number J is 60, and the corresponding thresholds for the Li(2p) + H_2_(*v* = 1, *j* = 0) and Li(2p) + H_2_(*v* = 0, *j* = 1) reactions are 0.43 and 0.93 eV, respectively. Therefore, the upper limit of the collision energy in the calculations of ICSs and DCSs is set as 0.43 eV. The total ICSs of the Li(2p) + H_2_ → LiH + H reaction with different initial rovibrational states of H_2_ molecule as a function of collision energy are depicted in Fig. 3. The ICSs of the title reaction with H_2_(*v* = 0, *j* = 0) and H_2_(*v* = 0, *j* = 1) are quite lower than the one of H_2_(*v* = 1, *j* = 0) over the whole collision energy range. At low collision energies, the ICS for H_2_(*v* = 1, *j* = 0) starts from a high value and then decreases rapidly with increasing collision energy, which is a typical feature of barrierless reactions. Due to the fact that the potential energy of Li(2p) + H_2_(*v* = 1, *j* = 0) reactants is higher than the potential energy of LiH(*v*ʹ = 0, 1) + H products as we observed in Fig. 1. In addition, we have found that the reaction process of the Li(2p) + H_2_(v = 1, j = 0) reaction is similar to the reaction processes of N(^2^D) + H_2_ and O(^1^D) + H_2_ reactions^23^. At low collision energies (*E*_col_ < 0.05 eV), the Li(2p) + H_2_(*v* = 1, *j* = 0) reaction is dominated by insertion mechanism leading to a long-lived intermediate complex which make a significant contribution to the oscillations in the ICS. At relatively high collision energies (*E*_col_ > 0.05 eV), the available energy is sufficient large for the reactants to surmount the centrifugal barrier and escape the potential well within a short time interval. The Li(2p) + H_2_(*v* = 1, *j* = 0) reaction is dominated by direct abstraction mechanism resulting in the absence of oscillations in the ICS over the collision energies of 0.05 to 0.43 eV.

In contrast, the ICSs for the Li(2p) + H_2_(*v* = 0, *j* = 0, 1) reactions increase monotonically with an increase of collision energy. Furthermore, it is observed that the rotational excitation of H_2_ molecule has small influence on the ICSs. Clearly, the vibration excitation of the H_2_ molecule has greater impact on the ICSs than the rotation excitation of H_2_ molecule, which is caused by the fact that the internal energy of H_2_(*v* = 1, *j* = 0) molecule is much larger than that of H_2_(*v* = 0, *j* = 1). To get information about the vibrational states of LiH products, the products vibrationally resolved ICSs of the Li(2p) + H_2_(*v* = 1, *j* = 0) → LiH(*v*′ = 0, 1, 2, 3) + H reaction are also presented in Fig. 3. However, the products vibrationally resolved ICSs of the Li(2p) + H_2_(*v* = 0, *j* = 0, 1) reactions are not presented in this figure due to the fact nearly all of the LiH products of the two reactions distribute in vibrational ground state. At low collision energies (*E*_col _≤ 0.05 eV), a few oscillations appears on the ICSs curves of LiH(*v*′ = 0, 1), which are similar to the results observed from the Ne + H_2_^+^reaction^24, 25^. In addition, we found the reaction path of the Ne + H_2_^+^ → NeH^+^ + H reaction is similar to the one of the title reaction. The oscillations on the ICSs of the Li(2p) + H_2_(*v* = 1, *j* = 0) reaction are probably induced by the resonances arising from of unstable H-Li-H complex, which resembles to the situation found in the Ne + H_2_^+^ → NeH^+^ + H reaction^24^.

### Vibrational and rotational state distributions

**Figure 4:**
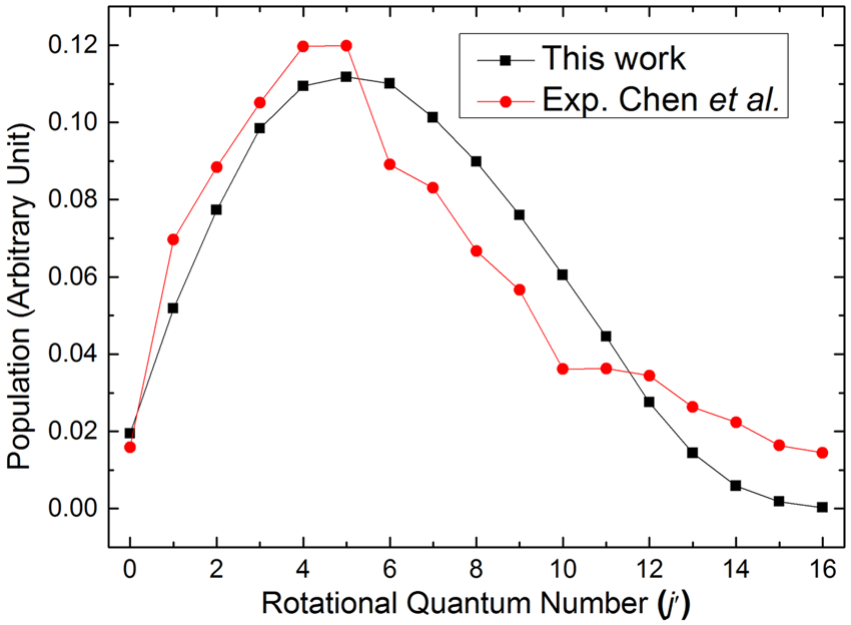
Comparison of rotational state distributions of the LiH products for the Li(2p) + H_2_(*v* = 0, *j* = 1) reaction between the theoretical and experimental results over the whole calculated collision energies (0.0025 < *E*_col_/eV < 0.43)^18^.

To verify the reliability of our dynamic results, a comparison for the rotational state distribution of the LiH products of the Li(2p) + H_2_(*v* = 0, *j* = 1) reaction between the theoretical and experimental results is presented in Fig. 4 ^18^. In order to make the theoretical results conform to the actual situation, the Maxwell speed distribution is considered in the theoretical results. The arbitrary unit is employed in the experiment, thus it is necessary to scale the experiment data to our theoretical results. In this work, both the sums of relative rotational populations of experimental and theoretical results are set to one. As can be seen from Fig. 4, the rotational state distribution of the theoretical results is in reasonable agreement with the experimental results, except for the range in which the rotational quantum number *j* is larger than 6. Owing to the fact that the H_2_ molecule is consisted of about 67% H_2_(*v* = 0, *j* = 1), 22% H_2_(*v* = 0, *j* = 3) and a small amount of H_2_(*v* = 0, *j* = 0, 2) molecules in Chen’s experiment, however, only the case with H_2_(*v* = 0, *j* = 1) was involved in the theoretical results of Fig. 4. In general, the trend of theoretical results is consistent with the experimental one, which validating the accuracy of our calculations.

**Figure 5:**
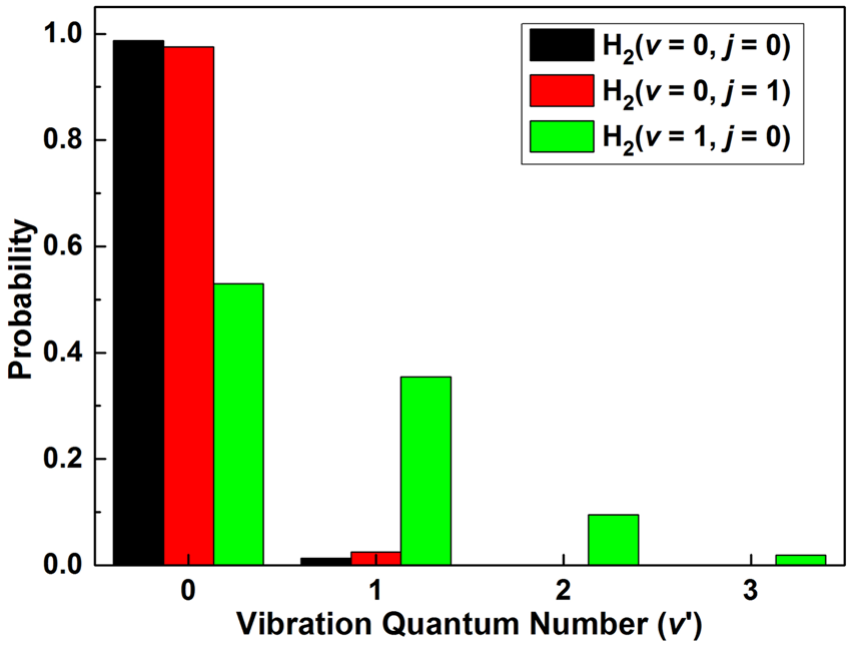
Vibrational state distributions of LiH molecules in the Li(2p) + H_2_ → LiH + H reaction for different initial rovibrational states of H_2_ molecule over the whole calculated collision energies (0.0025 < *E*_col_/eV < 0.43).

**Figure 6:**
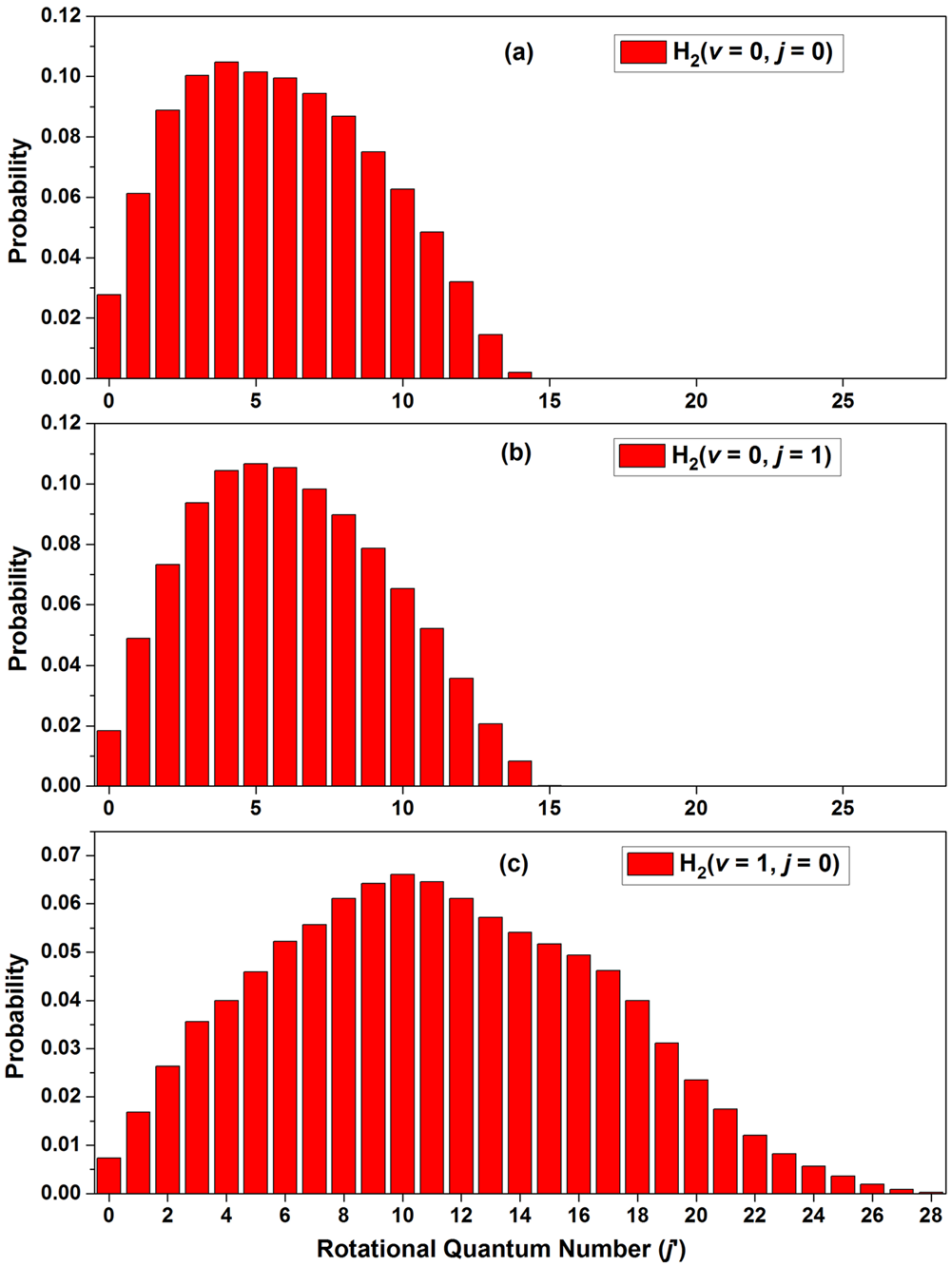
Rotational state distributions of LiH molecules in the Li(2p) + H_2_ → LiH + H reaction for different initial rovibrational states of H_2_ molecule over the whole calculated collision energies (0.0025 < *E*_col_/eV < 0.43). (**a**) H_2_(*v* = 0, *j* = 0); (**b**) H_2_(*v* = 0, *j* = 1); (**c**) H_2_(*v* = 1, *j* = 0).

In order to gain detailed information about the internal state distributions of the LiH product molecule, the vibrational state and rotational state distributions of LiH molecule are depicted in Figs 5 and 6, respectively. Figure 5 clearly shows that nearly all LiH product distributed in *v*′ = 0 state for the Li(2p) + H_2_(*v* = 0, *j* = 0, 1) reactants indicate that the rotational excitation (*j* = 1) of H_2_ molecule has a little influence on the vibrational state distributions of LiH product. At the same time, only a small fraction of LiH products distribute in *v′* = 1 state for the Li(2p) + H_2_(*v* = 0, *j* = 0, 1). Over the whole calculated collision energies (0.0025 ≤ *E*_col_/eV ≤ 0.43), the LiH molecule cannot be excited to higher vibrational level than *v′* = 1 for the Li(2p) + H_2_(*v* = 0, *j* = 0, 1) reactions. In contrast, the product LiH molecule distribute in four vibrational levels (from *v′* = 0 to *v′* = 3) for the Li(2p) + H_2_(*v* = 1, *j* = 0) reaction. More specifically, the LiH products are mainly generated in the ground vibrational state and the vibrational state distributions decrease gradually with the increase of vibrational quantum number. The vibrational state distribution of LiH products can be interpreted by taking into account the energy gap between reactants and products. As shown in Fig. 1, the product channel to highly excited vibrational state LiH molecule can be opened easily for the Li(2p) + H_2_(*v* = 1, *j* = 0) reactants with increasing collision energy. However, the product channel LiH(*v*′ = 2) + H is accessible for the Li(2p) + H_2_(*v* = 0, *j* = 1) reactants until the collision energy larger than 0.52 eV. In addition, the potential well located in the reaction path has important effect on the energy distribution of products. There are lots of bound and quasi-bound states in the intermediate complex, which is conducive to the distribution of available energy on the rovibrational states of product.

To obtain detailed information about the energy distribution, the rotational state distributions of LiH molecules are shown in Fig. 6 as a function of collision energies in the range of 0.0025 to 0.43 eV. The shape of the rotational state distributions for the Li(2p) + H_2_(*v* = 0, *j* = 0) reaction is similar to that of Li(2p) + H_2_(*v* = 0, *j* = 1) reaction. Moreover, the peaks of the rotational state distribution of LiH product for the reaction with H_2_(*v* = 0, *j* = 0) and H_2_(*v* = 0, *j* = 1) are *j′* = 4 and *j′* = 5, respectively. Comparing the middle panel with the bottom panel of Fig. 6, we can see that the upper limit of rotational quantum number of LiH product for the Li + H_2_(*v* = 0, *j* = 1) reaction is *j′* = 15, while the one for the Li + H_2_(*v* = 1, *j* = 0) reaction is *j′* = 28. Another important feature in bottom panel of Fig. 6 is that the peak of the rotational distribution of LiH product is *j′* = 10 which is larger than these of the Li(2p) + H_2_(*v* = 0, *j* = 0, 1) reactions. In all cases, the percentage of the rotational level population of the LiH product increases rapidly before reaching peak, and then decreases gradually with the increase of the rotational quantum number. For the reaction Li + H_2_(*v* = 1, *j* = 0), the reaction is dominated by abstraction mechanism gradually with the increase of collision energy, which means this reaction prefer to form a H-H-Li intermediate complex rather than a H-Li-H intermediate complex. In this this case, the energy released by the H_2_(*v* = 1, *j* = 0) more likely to transform to the rotational energy of LiH.

### Differential cross sections

**Figure 7:**
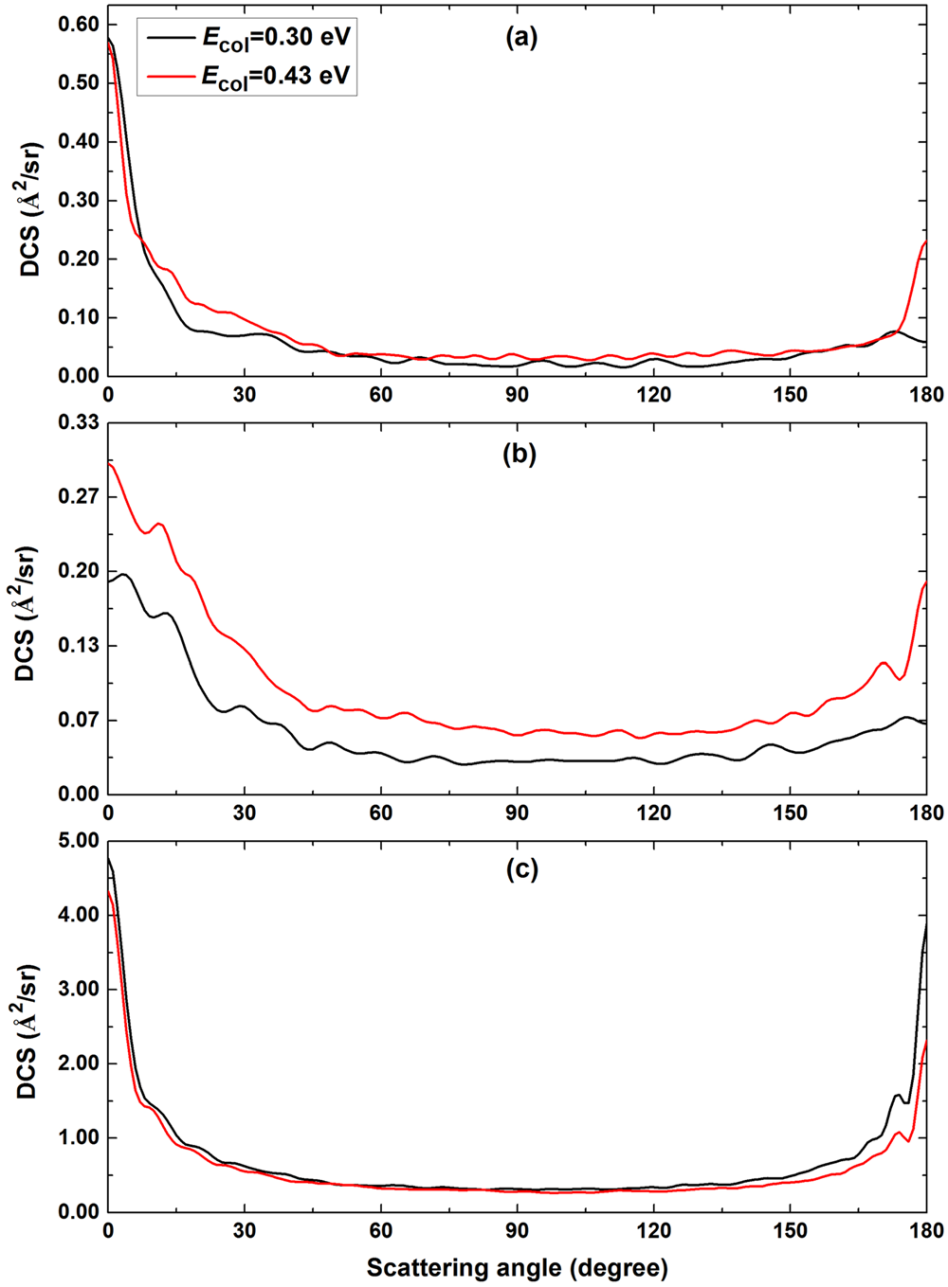
Total DCSs of the Li(2p) + H_2_ → LiH + H reaction at two collision energies of 0.30 and 0.43 eV. (**a**) H_2_(*v* = 0, *j* = 0); (**b**) H_2_(*v* = 0, *j* = 1); (**c**) H_2_(*v* = 1, *j* = 0).

Figure 7 shows the DCSs for the title reaction at two collision energies of 0.30 and 0.43 eV. The three panels from (a) to (c) correspond to different initial rovibrational states of H_2_ reactant. Despite the variation trends of the DCSs with increasing collision energy are different, the DCSs in the three panels show a forward scattering (0° < *θ* < 60°) tendency. From the upper panel of Fig. 7, it is clearly seen that the LiH product molecules for the Li(2p) + H_2_(*v* = 0, *j* = 0) reaction are scattered mainly in the forward hemisphere at the both collision energies. Only few LiH products scatter at the sideways (60° < *θ* < 120°). In addition, it is observed that more and more the LiH product molecules prefer to scatter at the backward hemisphere (120° < *θ* < 180°) with increasing collision energy. As shown in the middle panel of Fig. 7, the forward and the sideways scattering are enhanced gradually with increasing collision energy. At the same time, the increased degree of LiH molecules scattering at backward direction are more than these scattering at forward direction. Since the energy level of the LiH(*v*′ = 0) + H products is higher than the energy levels of Li(2p) + H_2_(*v* = 0, *j* = 0, 1) reactants, long-lived complexes cannot be converted into LiH + H products efficiently at low collision energy of 0.3 eV. In this case, the products are yielded by reactions with single collision and the energies released by H_2_(*v* = 0, *j* = 0, 1) have a small influence on the scattering direction of LiH molecule, the scattering direction of product molecule is mainly determined by the velocity of incoming atom. This mechanism leads to that the DCSs of the Li(2p) + H_2_(*v* = 0, *j* = 0, 1) reactions are governed by forward scattering at low collision energies. At collision energy of 0.43 eV, more and more LiH + H products are formed by long-lived complexes which will reduce the effect of velocity of the incoming atom by its chaotic rovibration motions. This mechanism leads to the angular distribution isotropic, i.e., the DCS symmetric with respect to θ = π/2^26, 27^. It appears as the significant enhancement of the backward peaks of the DCSs for the Li + H_2_(*v* = 0, *j* = 0, 1).

**Figure 8:**
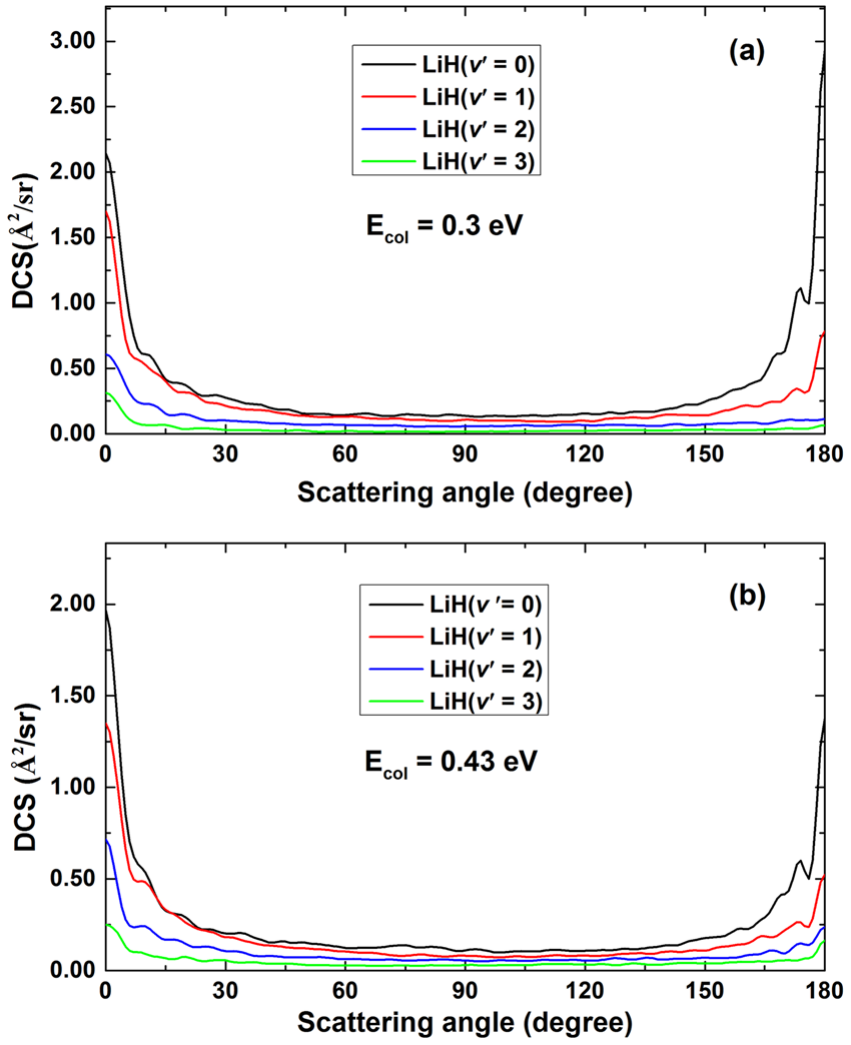
The products vibrationally resolved DCSs of the Li(2p) + H_2_(*v* = 1, *j* = 0) → LiH(*v*′ = 0, 1, 2, 3) + H reactions at two collision energies.

In contrast to the influence of rotational excitation of the H_2_ on ICSs, the rotational excitation of the H_2_ reactant molecule has a significant effect on the DCSs of the title reaction. Comparing the upper panel with the middle panel of Fig. 7, it is found that the difference among the forward, sideways and backward scattering is reduced when the H_2_ reactant molecule is excited to the first rotationally excited state from ground state. The trend of forward scattering peak of LiH molecule as angular momentum *j* increase of reactant molecule is similar to the results obtained from the D^+^ + H_2_ reaction^27^. The polarization peaks (peak close to 0° an 180°) are contributed by the collisions with *K* = *K*_0_ = 0, where *K* and *K*_0_ are the projection quantum number of the diatom rotational state onto the initial and final relative translational velocity vectors, respectively. The statistical weight [1/(2*j*_0_ + 1)] of the collisions with *K* = *K*_0_ = 0 decreases as the increases of the angular momentum of H_2_^27^, thus the forward and backward scattering peaks decrease with the increase of the rotational excitation of H_2_. In comparison with the middle panel of Fig. 7, the bottom panel exhibits opposite behavior. The DCSs for the Li(2p) + H_2_(*v* = 1, *j* = 0) reaction decrease with the increase of the collision energy, especially the backward scattering decrease rapidly as the collision energy increases. Obviously, the DCSs of the Li + H_2_(*v* = 1, *j* = 0) reaction is dominated by the forward scattering with increasing collision energy. It reveals that the direct mechanism becomes more prevalent for the title reaction with H_2_(*v* = 1, *j* = 0) with increasing collision energy. In order to gain additional insight into the reaction dynamics of the title reaction, the products vibrationally resolved DCSs of the Li(2p) + H_2_(*v* = 1, *j* = 0) → LiH(*v*′ = 0, 1, 2, 3) + H reaction are presented in Fig. 8. The products vibrationally resolved DCSs of the Li(2p) + H_2_(*v* = 0, *j* = 0, 1) reactions are not presented in this work since the LiH products of these two reactions are almost entirely distributed in vibrational ground state. For the results of LiH(*v*′ = 0), both forward and backward scattering can be observed at the two collision energies of 0.3 and 0.43 eV. It is notable that the scattering angle corresponding to the maximum of the DCS of LiH(*v*′ = 0) is changing from *θ* = 180° to *θ* = 0° with the increase collision energy. Comparing the DCS of LiH(*v*′ = 1) in Fig. 8(a) with the one in Fig. 8(b), the degree of forward scattering is enhanced as the collision energy increases. It is due to that the direct mechanism is more prevalent for the Li(2p) + H_2_(*v* = 1, *j* = 0) → LiH(*v*′ = 1) + H reaction at relatively high collision energies. The backward scatterings of the DCSs for LiH(*v*′ = 2, 3) are more pronounced at collision energy of 0.43 eV than these at collision energy of 0.3 eV. This reveal that more and more long-lived complexes transform into highly excited vibrational LiH(*v*′ = 2, 3) products. In other words, the direct and indirect mechanism exist in the Li(2p) + H_2_(*v* = 1, *j* = 0) → LiH + H reaction simultaneously over the collision energies range of 0.3–0.43 eV.

**Figure 9:**
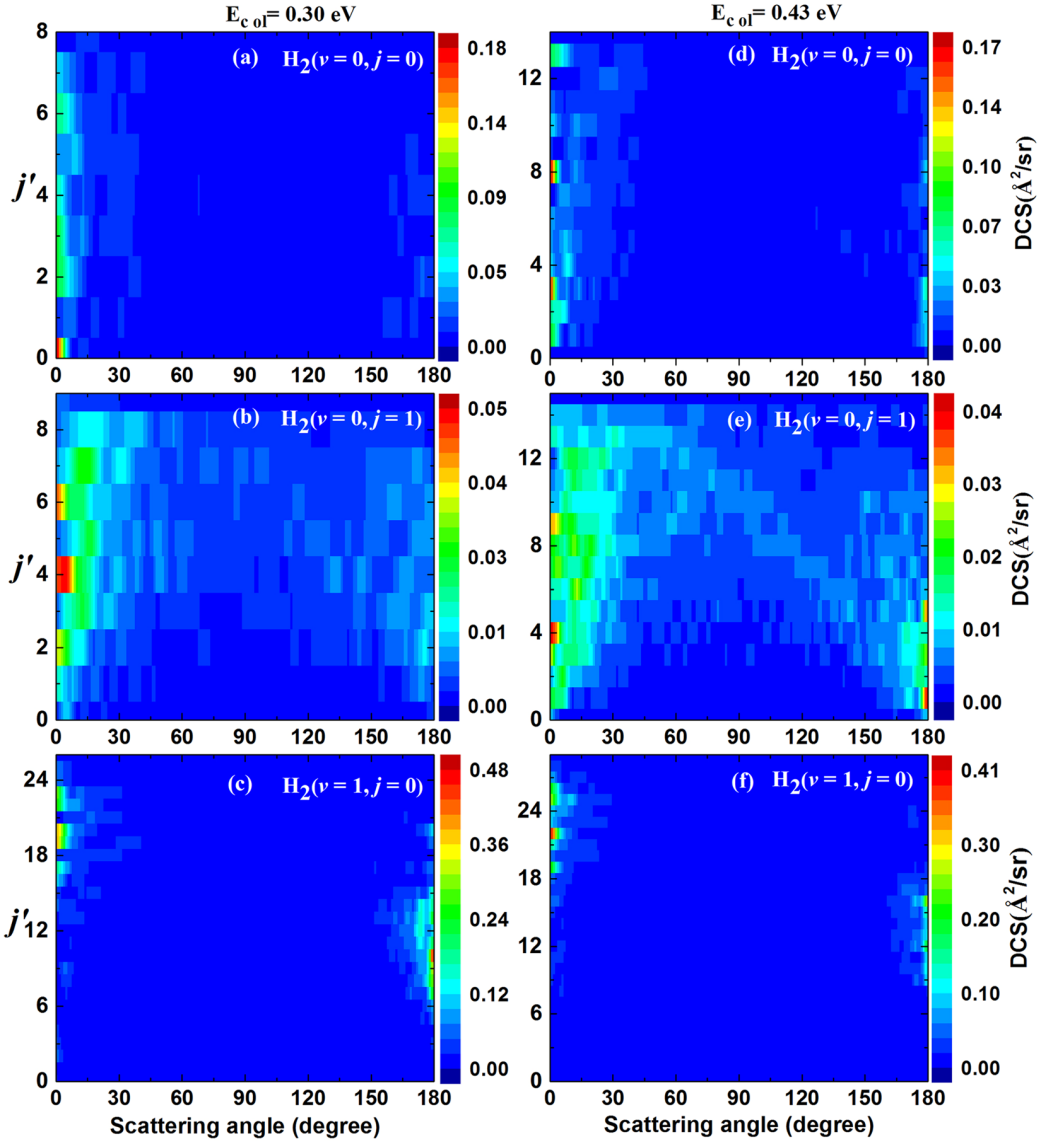
State-to-state DCSs(Å^2^/sr) of the Li(2p) + H_2_ → LiH + H reaction for different initial rovibrational states of H_2_ molecule. Left panels: at collision energy of 0.30 eV; right panels: at collision energy of 0.43 eV.

To obtain deeply understanding about the reaction mechanism of the title reaction, the state-to-state DCSs at two collision energies of 0.30 and 0.43 eV are calculated. Only the DCSs of LiH(*v′* = 0) products are presented in Fig. 9 due to the fact that most of the LiH product molecules are distributed in ground vibrational state. The left and right panels show the DCSs for *E*_col_ = 0.30 eV and *E*_col_ = 0.43 eV, respectively. The left panels from (a) to (c) correspond to the reactant molecule at H_2_(*v* = 0, *j* = 0), H_2_(*v* = 0, *j* = 1) and H_2_(*v* = 1, *j* = 0), and the same to the right panels from (d) to (f). By comparing Fig. 9(a) with Fig. 9(d), it can be concluded that the DCSs for LiH(*v*′ = 0) at the two collision energies are dominated by forward scattering, and the backward scattering at collision energy of 0.30 eV can be ignored. In addition, the backward scattering peak for *E*_col_ = 0.43 eV is contributed by lowly excited rotational LiH products. For the Li(2p) + H_2_(*v* = 0, *j* = 0) reaction, long-lived complexes cannot be transformed into LiH + H products efficiently at collision energy of 0.3 eV. With increasing collision energy, a small number of long-lived complexes with low internal energy can be converted into products, thus the backward scattering is mainly contributed by rotationally lowly excited LiH molecules. It is clearly seen from the upper panels and the middle panels of Fig. 9 that the sideways scattering is enhanced while the H_2_ reactant is excited to first rotationally excited state.

**Figure 10:**
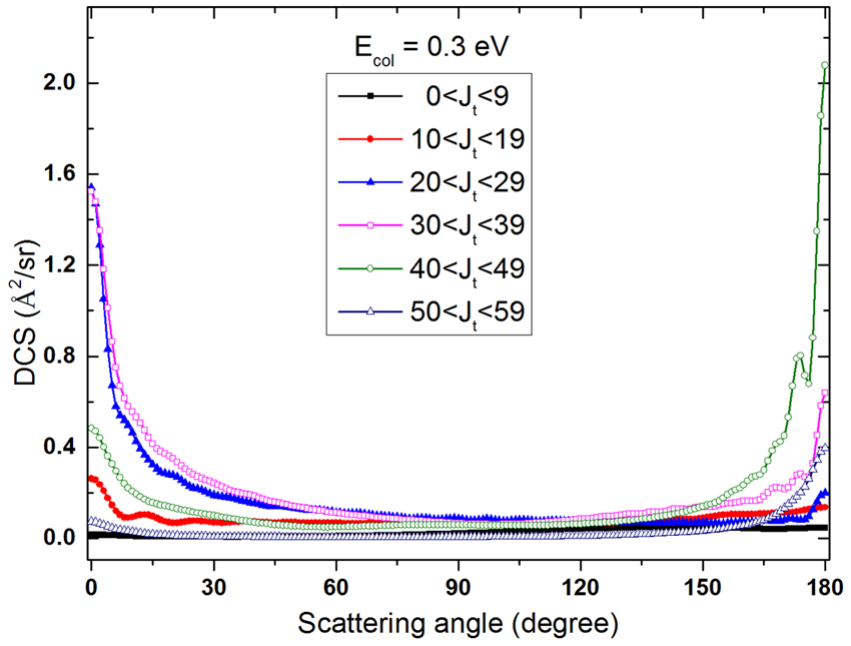
DCSs of the Li(2p) + H_2_(*v* = 1, *j* = 0) → LiH + H reaction for different total angular momentum J at collision energy of 0.30 eV.

As can be seen from the bottom panels of Fig. 9, the forward and backward peaks of the DCSs distributed in different range of *j*′. The forward peaks distribute in large rotational quantum number *j*′ and the backward peaks distribute in relatively small rotational quantum number *j*′. The state-to-state DCSs exhibit an asymmetrical feature, which is a typical characteristic of direct reaction. The backward peaks of the DCSs are mainly contributed by the reactive collisions with large total angular momentum and the forward peaks of the DCSs are mainly contributed the reactive collision with small total angular momentum. To further explain the reaction mechanism of the Li(2p) + H_2_(*v* = 1, *j* = 0) → LiH + H reaction more clearly, the differential cross sections (DCSs) of the Li(2p) + H_2_(*v* = 1, *j* = 0) → LiH + H reaction for different total angular momentum presented have been analyzed in Fig. 10. It is shown that the DCSs forward peaks are mainly contributed by reactive collisions with small total angular momentum (J ≤ 39). At the same time, the DCSs backward peaks are mainly contributed by reactive collisions with relatively larger total angular momentum (J ≥ 40). The forward and backward peaks are distributed in different range of total angular momentum, which means the Li(2p) + H_2_(*v* = 1, *j* = 0) → LiH + H reaction is dominated by direct reaction mechanism rather than complex-forming mechanism.

The Li atom collides with the H_2_ molecule with large total angular momentum, and the centrifugal barrier makes the reaction path of the Li(2p) + H_2_(*v* = 1, *j* = 0) reaction similar to the one which take place on a repulsive PES. The H_2_(*v* = 1, *j* = 0) molecule start to release its internal energy till the Li atom close to the H_2_ molecule in this case. Most of the energy released by the H_2_(*v* = 1, *j* = 0) molecule turns into translational energy of products, and only a little energy released by reactants converted into the internal energy of LiH products. Therefore, the LiH products with relatively low rotational states exhibit evident backward scattering tendency. In contrast, the forward scattering LiH products with rotationally highly excited states are mainly contributed by these reactions with small total angular momentum. The H_2_(*v* = 1, *j* = 0) molecule start to release its internal energy when the Li atom towards to H_2_ molecule in this case. Most of the internal energy of the reactants are converted into the rotational energy of LiH products, hence, the velocity of the LiH molecules nearly parallel to the velocity direction of the incoming Li atom. This mechanism has mainly contributed to forward scattering tendency of rotationally highly excited LiH molecule. These behaviours are in agreement with the analyses of Polanyi^28^.

## Discussion

The influence of the rovibrational excitation of H_2_ molecule on the Li(2p) + H_2_ → LiH + H has been investigated using a set of diabatic PESs constructed by our group^22^. The vibrational excitation of H_2_ reactant has greater influence than the rotational excitation on the reaction probability and ICS of the title reaction due to the fact that the energy released by the vibrationally excited H_2_(*v* = 1, *j* = 0) is larger than the one released by the rotationally excited H_2_(*v* = 0, *j* = 1). The threshold energy on the curves of reaction probability and ICS are eliminated when the H_2_ reactant molecule is excited to the first excited vibrational states, which indicate the endothermicity of the reaction can be overcome by the energy released from the internal energy of H_2_(*v* = 1, *j* = 0) molecule. The vibrational and rotational state distributions of LiH product reveal that the internal energy of the H_2_(*v* = 1, *j* = 0) reactant could be transformed into internal energy of LiH product effectively. The results of DCSs show that the angular distribution of the LiH product molecule is dominated by forward scattering. The rotational excitation, vibrational excitation and collision energy of reactants have different effects on the DCSs. Comparing the DCSs of the Li(2p) + H_2_(*v* = 0, *j* = 0) reaction with the DCSs of the Li(2p) + H_2_(*v* = 0, *j* = 1) reaction, the rotational excitation of H_2_ molecule enhances the sideways and backward scattering of LiH products. The microscopic mechanism of the Li(2p) + H_2_(*v* = 1, *j* = 0) reaction is very complicated. More specifically, the direct reaction mechanism dominates the Li(2p) + H_2_(*v* = 1, *j* = 0) → LiH(*v* = 0) + H reaction, and the direction of the vibrationally lowly excited LiH(*v* = 0) product is very sensitive to the total angular momentum of the reactants. In addition, both direct and indirect reaction mechanism exist simultaneously for the reactions with vibrationally highly excited LiH(*v* = 1, 2, 3) products. Otherwise, for the reaction with vibrationally excited H_2_ molecule, the forward scattering of LiH products is enhanced with increasing collision energy. In general, the scattering behaviour of the title reaction can be attributed by a competition between the two different reaction mechanisms.

## Methods

### Diabatic Potential Energy Surfaces

The diabatic PESs (HYLC PESs) employed here are constructed based on 30,796 high-level *ab initio* energy points which are calculated by multi-reference configuration interaction calculations using basis sets of quadruple zeta quality. The diabatic potential energies are obtained from the diabatization scheme based on transition dipole momentum operators. The neural network method was used for fitting the diabatic PESs. As far as we know, it is the most accurate diabatic PESs for the title reaction to date. Figure 1 depicts the schematic energy diagram of the title reaction according to the HYLC PESs. There is a potential well about 0.8 eV along the reaction path due to the surface crossing between the two coupled states. The potential well located in the entrance channel has a great influence on the reaction dynamics of title reaction, especially at the low collision energy range. More details of the HYLC PES can be found in Ref. 22.

### Dynamical Calculations

The TDWP method has been successfully applied to study the reaction dynamics in many reactive systems^29–35^. The theory of the TDWP method has been extensively described in previous work^36, 37^. Here, we only introduce the outline of the TDWP method. The TDWP method employed in this study is developed by Sun *et al*., which only requires the wave-packet propagate in reactant Jacobi coordinates to extract the *S*-matrix information. This method is fairly effective in dealing with the reaction, which takes place over two coupled PESs, such as the Cl + H_2_ reaction^32^. In the body fixed representation, the Hamiltonian of the Li(2p) + H_2_ system can be written as

1 $$\hat{H}={\hat{H}}_{0}+\hat{U},$$

where

2 $${\hat{H}}_{0}=-\frac{{\hslash }^{2}}{2{\mu }_{R}}\frac{{\partial }^{2}}{\partial {R}^{2}}+\hat{h}(r),$$

3 $$\hat{U}=\frac{{(\hat{J}-\hat{j})}^{2}}{2{\mu }_{R}{R}^{2}}+\frac{{\hat{j}}^{2}}{2{\mu }_{r}{r}^{2}}+{\hat{V}}^{d}={\hat{V}}_{rot}+{\hat{V}}^{d}.$$

Here,$\,\hat{h}(r)$ is the diatom reference potential. *R* is the vector from Li atom to the mass center of the H_2_ molecule and *r* is the vibrational vector of the H_2_ molecule, ${\mu }_{R}$ and ${\mu }_{r}$ acre the corresponding reduced masses. *J* and *j* are total angular momentum operator of the LiH_2_ system and rotational angular momentum operator of reactant diatomic molecule, respectively. ${\hat{V}}^{d}$ is a 2 × 2 matrix which represents the potential energy of the LiH_2_ system. The split operator method was employed in the propagation of the wave function:

4 $$[\begin{array}{c}{\psi }_{1}(t+{{\rm{\Delta }}}_{t})\\ {\psi }_{2}(t+{{\rm{\Delta }}}_{t})\end{array}]={e}^{-i{H}_{0}{{\rm{\Delta }}}_{t}/2}{e}^{-i{V}_{tot}{{\rm{\Delta }}}_{t}/2}{e}^{-i[\begin{array}{cc}{V}_{11}^{d} & {V}_{12}^{d}\\ {V}_{21}^{d} & {V}_{22}^{d}\end{array}]{{\rm{\Delta }}}_{t}}{e}^{-i{V}_{tot}{{\rm{\Delta }}}_{t}/2}{e}^{-i{H}_{0}{{\rm{\Delta }}}_{t}/2}[\begin{array}{c}{\psi }_{1}(t)\\ {\psi }_{2}(t)\end{array}].$$

The diabatic potential matrix ${\hat{V}}^{d}$ can be transformed to adiabatic potential matrix ${\hat{V}}^{a}$ using the diabatic-to-adiabatic transformation matrix *T*

5 $$[\begin{array}{cc}{V}_{1}^{a} & 0\\ 0 & {V}_{2}^{a}\end{array}]=T[\begin{array}{cc}{V}_{11}^{d} & {V}_{12}^{d}\\ {V}_{21}^{d} & {V}_{22}^{d}\end{array}]{\tilde{T}}^{+}.$$

According to the Eq. (5), the Eq. (4) can be written as

6 $$[\begin{array}{c}{\psi }_{1}(t+{{\rm{\Delta }}}_{t})\\ {\psi }_{2}(t+{{\rm{\Delta }}}_{t})\end{array}]={e}^{-\frac{i{H}_{0}{{\rm{\Delta }}}_{t}}{2}}{e}^{-\frac{i{V}_{tot}{{\rm{\Delta }}}_{t}}{2}}T[\begin{array}{cc}{e}^{-i{V}_{1}^{a}{{\rm{\Delta }}}_{t}} & 0\\ 0 & {e}^{-i{V}_{2}^{a}{{\rm{\Delta }}}_{t}}\end{array}]{\tilde{T}}^{+}\times {e}^{-i{V}_{tot}{{\rm{\Delta }}}_{t}/2}{e}^{-i{H}_{0}{{\rm{\Delta }}}_{t}/2}[\begin{array}{c}{\psi }_{1}\,(t)\\ {\psi }_{2}\,(t)\end{array}],$$

The reactant-coordinate-based method is used to extract the state-to-state *S*-matrix. The state-to-state reaction probability is obtained by

7 $${P}_{vj\leftarrow {v}_{0}{j}_{0}}^{J}=\frac{1}{2{j}_{0}+1}\sum _{K,{K}_{0}}{|{S}_{vjK\leftarrow {v}_{0}{j}_{0}{K}_{0}}^{J}|}^{2}.$$

The state-to-state ICS is given by

8 $${{\rm{\sigma }}}_{vj\leftarrow {v}_{0}{j}_{0}}=\frac{{\rm{\pi }}}{(2{j}_{0}+1){{\rm{k}}}_{{v}_{0}{j}_{0}}^{2}}\sum _{K}\sum _{{K}_{0}}\sum _{J}(2J+1){|{{\rm{S}}}_{vjK\leftarrow {v}_{0}{j}_{0}{K}_{0}}^{J}|}^{2},$$

The state-to-state DCS can be calculated by the following equation:

9 $$\frac{d{\sigma }_{vj\leftarrow {v}_{0}{j}_{0}}(\theta ,E)}{d{\rm{\Omega }}}=\frac{1}{(2{j}_{0}+1)}\sum _{K}\sum _{{K}_{0}}{|\frac{1}{2i{k}_{{v}_{0}{j}_{0}}}\sum _{J}(2J+1){d}_{K{K}_{0}}^{J}(\theta ){S}_{vjK\leftarrow {v}_{0}{j}_{0}{K}_{0}}^{J}|}^{2},$$

in which the *θ* is the scattering angle between the incoming Li(2p) + H_2_ reactants and the scattered LiH + H products. The Wigner function ${d}_{K{K}_{0}}^{J}(\theta )$ is used for calculating the reduced rotation matrix element. In order to consider the effect of the centrifugal potential operator properly, the full Coriolis-coupled are involved in the TDWP calculations. In this study, the rovibrational states of H_2_ molecule are set as (*v* = 0, *j* = 0), (*v* = 0, *j* = 1) and (*v* = 1, *j* = 0) for investigating the influence of the rovibrational excitation. The numerical parameters used in this calculations aresummarized in Table 1, the different numerical parameters for different initial states of H_2_ molecules are also presented in this table.

**Table 1:** Numerical Parameters used in the TDWP calculations.

Grid/basis range and size	*R* ∈ [0.01 a.u., 15.0 a.u.], N_*R*_ = 149
	*r* ∈ [0.01 a.u., 15.0 a.u.], N_*R*_ = 149
	*j*_min_ = 0 ~ *j*_max_ = 69, *N*_*j*_ = 69 over [0°,180°]
Initial wave -packet	*R*_0_ = 10.0 a.u.
$\exp [-\frac{(R-{R}_{0})}{2{\Delta }_{R}^{2}}i{k}_{0}R]$	Δ_*R*_ = 0.08 a.u.
	*k*_0_ = (2*E*_0_ *μ*_*R*_)^1/2^with *E*_0_ = 0.5 eV
Absorption functions for H_2_(*v* = 1, *j* = 0)	exp[−0.0192 × Δ_*t*_(*r* − 12.5)^2^], for 12.5 ≤ *r* ≤ 15.0
	exp[−0.016 × Δ_*t*_(*R* − 12.5)^2^], for 12.5 ≤ *R* ≤ 15.0
Absorption functions for H_2_(*v* = 0, *j* = 0, 1)	exp[−0.0192 × Δ_*t*_(*r* − 12.5)^2^], for 12.5 ≤ *r* ≤ 15.0
	exp[−0.0144 × Δ_*t*_(*R* − 12.5)^2^], for 12.5 ≤ *R* ≤ 15.0
Total propagation time	40000 a.u.
Time step	15 a.u.
Highest J Value	60
